# Tumours and tumour-like lesions of the lower face at Korle Bu Teaching Hospital, Ghana – an eight year study

**DOI:** 10.1186/1477-7819-5-48

**Published:** 2007-05-07

**Authors:** Grace EA Parkins, George Armah, Patrick Ampofo

**Affiliations:** 1 Department of Oral and Maxillofacial Surgery, University of Ghana Dental School, College of Health Sciences, Korle-Bu, Accra, Ghana; 2 Noguchi Memorial Institute for Medical Research, College of Health Sciences, University of Ghana, Legon, Accra, Ghana

## Abstract

**Background:**

The oro-facial region including the jawbones, the maxilla and mandible and related tissues can be the site of a multitude of neoplastic conditions. These tumours have a predilection for the entire facial region; however, odontogenic tumours tend to affect the mandible more than the maxilla, especially, in West African children. We report results from a retrospective study spanning eight years on the frequency, clinical presentation, sites and character of lower face tumours seen in the main referral hospital in Ghana.

**Patients and methods:**

Records of consecutive patients of all age and sex seen by the first author's team at the Department of Oral and Maxillofacial Surgery, Korle-Bu Teaching Hospital with tumours affecting the lower part of the face from January 1996 to December 2003 were retrieved, coded and entered into a database. The data were then analyzed by age, sex, presenting signs and symptoms, site of lesion, and their histology.

**Results:**

A total of 394 patients with oro-facial swellings were retrieved from the registry out of which 210 had lower face tumour and tumour-like lesions. The complete data set was obtained for 171 patients, comprising 99 (58%) males and 72 (42%) females. The most common clinical presenting features were mandibular facial swelling (63%), intra-oral swelling (55%), pain (41%) and ulceration (29%). The tumours were predominantly found in the right (43%), anterior (19%) and left (18%) aspects of the lower face. The remainder making up 20% were found in the floor of the mouth, tongue and lips. Seventy eight (45.6%) of the patients presented with lesions that were classified as malignant of which 54 (62%) were diagnosed as squamous cell carcinoma (SCC). Sixty-two (36.3%) had benign odontogenic tumours and thirty-one (18.1%) had non-odontogenic tumour-like lesions. Fifty-four (62%) of malignant tumours were squamous cell carcinoma; 58 (93.6%) of the benign odontogenic tumours were classified as ameloblastoma. The mean age at presentation of all lesions was 40.4 years with over 50% of benign lesions in patients aged between 11 and 30 years. Malignant tumours were more commonly detected in patients between 41 and 70 years (63%).

**Conclusion:**

Tumours and tumour-like lesions of the lower face comprising the mandible, tongue and adjacent structures are a diverse group of neoplasm and are seen commonly in practice of Maxillofacial surgery. Both malignant and benign tumours are seen in the Ghanaian population. In the present study, SCC and ameloblastoma were the commonest malignant and benign odontogenic tumours seen respectively; the two representing more than 65% of all tumours.

## Background

The oro-facial region including the jawbones and related tissues can be the site of a multitude of neoplastic conditions [1]. Tumours affecting the lower face are common whilst those affecting the mid face are uncommon [2]. These tumours can be either malignant or benign. The malignant lesions usually found in the lower face include sarcomas of soft and hard connective tissue, carcinomas of the salivary glands, with SCC accounting for more than 90% of reported malignant tumours of the oral cavity and rarely melanomas [3,4]. Some of these cancers however are metastases from distant sites such as the breast, lungs, abdominal organs or even the prostate gland [5]. Benign lesions found in the lower face are odontogenic or non-odontogenic tumours, predominantly ameloblastoma [6].

The lower face is made up of the entire mandible supported by the covering soft tissue and encloses the tongue. The region is bounded superiorly by the mid face and posteriorly by the oropharynx. The paired submandibular and sublingual salivary glands, the minor salivary glands of the lower lip, muscles and structures of the floor of the mouth are included in this region. Inferiorly, the mandible forms a boundary for the anterior triangle of the neck, which is only separated from the posterior triangle by the sternocleidomastoid muscle [7]. These triangles contain lymph nodes into which malignant tumours spread primarily from the head and neck region and form channels through which malignancies also spread from lower parts of the body [7].

Thorough knowledge of regional anatomy is essential to understand the behaviour of both malignant and benign lesions of this region and the pattern of spread of tumours to the lymph nodes. Management of these tumours present a challenge due to their sizes at presentation in the West African region. There have been International reports on tumours of the jaws in the West African sub region, mainly from Nigeria, as well as other parts of the continent, but none from Ghana. The studies from Nigeria [8,9] show that odontogenic tumours have a predilection for the lower face while one study from Jordan found more non-odontogenic tumours in the mandible [10]. The Maxillofacial Surgery Department of the Korle Bu Teaching Hospital in Accra, the capital of Ghana, is the main referral hospital in the country and receives patients from the whole country as well as from the neighbouring countries of Ivory Coast, Burkina Faso and Togo, but predominantly from Southern Ghana. The aim of this study is to find the tumours and tumour-like lesions predominant in the lower face, their clinical and sites of presentation in all age groups. It is envisaged that results presented here will add to the depth of information on facial tumours in this region and in Africa at large.

## Patients and methods

Records of consecutive patients seen by the first author's team at the Department of Oral and Maxillofacial Surgery, Korle-Bu Teaching Hospital with tumours and tumour-like lesions affecting the lower face over a period of 8 years (1996–2003) were retrieved. The clinical presentations, sites of lesions, histopathology and demographic information were coded and double-entered into a computer database using the epi-info  software. The complete data set was analysed using the STATA (Stata Corp LP, USA) statistical software package.

## Results

**Figure 1:**
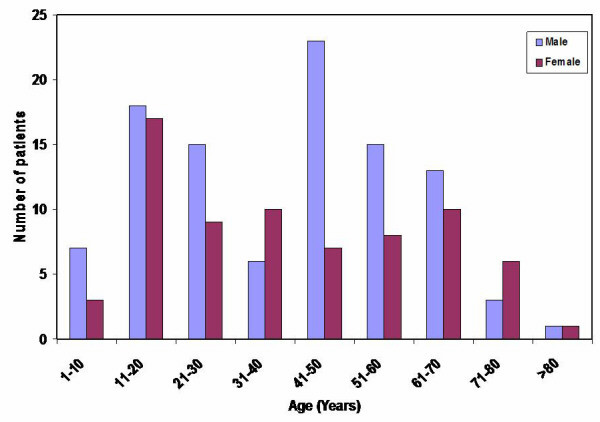
Age distribution of lower face tumours and tumour-like lesions (Korle-Bu Teaching Hospital, Accra (1996–2003).

A total of 394 with oro-facial swellings were retrieved from the registry out of which 210 had lower face tumour and tumour-like lesions, and a complete data set was obtained for 171 patients. The mean age at presentation was 40.4 years with the youngest and oldest patients being 4 and 95 years, respectively. Lesions were detected in all ages in both sexes, with males representing 57.9% (99/171) of all patients seen and the rest 42.1% (72/171) were females. The male to female ratio was 1.4:1. However, in males, lower face tumours and tumour-like lesions were commonly detected between the ages 41 and 50 years (23.2%) whilst in females it was very common in the younger age group of 11 to 20 years (23.6%), shown in Figure 1. Table 1 shows the most common presenting clinical features were mandibular facial swelling (63%), intra-oral swelling (55%), pain (41%) and ulceration (29%). Loosened and displaced teeth and enlarged lymph nodes were commonly associated with the malignant tumours (squamous cell carcinoma, the lymphomas and chondrosarcoma). Many patients had multiple presenting features; these included those listed above in addition to mobile, displaced or exfoliated teeth, enlarged or fixed submandibular or cervical lymph nodes, swollen tongue or lip (Table 1).

**Table 1:** Main presenting features

**Features**	**No. of cases (%)**
Mandibular facial swelling	108 (63)
Intra-oral swelling	94 (55)
Pain	70 (41)
Ulceration of oral cavity	50 (29)
Loose teeth	20 (12)
Displaced teeth	17 (10)
Enlarged lymph nodes	16 (9)
Swollen tongue	9 (5)
Swollen lower lip	8 (5)
Submandibular swelling	7 (4)
Enlarged cervical lymph nodes	7 (4)
Perforating ulcer	4 (2)
Exophytic growth of tongue	4 (2)
Necrotic ulcer	3 (2)
Severe trismus	2 (1)
Fixed tongue	2 (1)
Fixed submandibular lymph nodes	2 (1)
Enlarged bilateral submandibular lymph nodes	2 (1)
Bilobed swelling	1 (1)
Exfoliated teeth	1 (1)
Submental swelling	1 (1)

**Totals**	**428**

**Figure 2:**
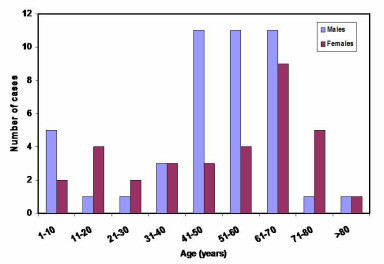
Age distribution of malignant tumours.

**Figure 3:**
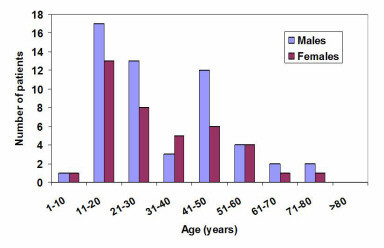
Age distribution of patients with benign tumours.

Tumours were sited at every aspect of the lower face. However, the right mandible was the most affected site with 73 (43%) of the tumours, followed by the anterior mandible (32, 19%) and the left mandible (30, 18%), shown in Table 2. About 20% were found on the floor of the mouth, tongue and lips. Some lesions were so large that they involved numerous sites. Seventy-eight (45.6 %) of these tumours were classified as malignant, sixty-two (36.3%) as benign odontogenic and thirty-one (18.1%) as benign non-odontogenic and tumour-like lesions. Malignant tumours were commonly detected in patients aged between 41 and 70 years (63%); whilst in the case of benign tumours, the frequency was higher in patients aged between 11 and 30 and between 41 and 50 years. Fifty-four (69.2%) of the malignant tumours were recorded in males and twenty-four (30.8%) in females; an M:F ratio of 2.3:1. Similarly, fifty-four (58.1%) and thirty-nine (41.9%) of the benign tumours were in males and females, respectively, ratio of 1.4:1. The mean age of occurrence of malignant and benign tumours were 49.7 ± 21.5 and 32.5 ± 16.9 years, respectively. The age distribution of the tumours seen during the period is shown in Figures 2 and 3.

**Table 2:** Site of lesion

**Site**	**No of Cases (%)**
Right mandible	73 (43)
Anterior mandible	32 (19)
Left mandible and/or ramus	30 (18)
Floor of mouth	19 (11)
Ventral surface of tongue	13 (8)
Lateral surface of tongue	13 (8)
Lower lip	12 (7)
Retromolar region	8 (5)
Buccal mucosa	7 (4)
Right submandibular region	7 (4)
Mental region of mandible	7 (4)
Dorsum of anterior tongue	6 (4)
Left submandibular region	5 (3)
Submental region	4 (2)
Posterior tongue	1 (1)

**Totals**	**237**

Fifty-four (69.2%) of the malignant tumours were diagnosed as SCC and fifteen (19%) as lymphoma. Other malignant lesions encountered were metastatic adenocarcinoma, chondrosarcoma, spindle cell carcinoma, mucoepidermoid carcinoma, ameloblastic carcinoma and plasmacytoma. Of the fifteen patients who suffered from lymphomas, eight had the Non-Hodgkin's type and seven, Burkitt's lymphoma. Patients with Burkitt's lymphoma had age range from 4 to 12 years whilst in the case of Non-Hodgkin's they were detected in older children and adults aged between 12 and 70 years.

**Figure 4:**
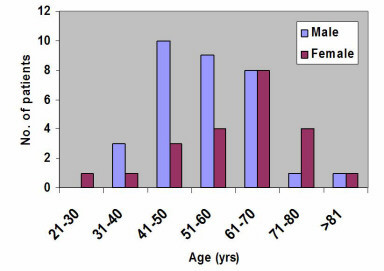
Age distribution of patients with squamous cell carcinoma.

Ninety-one percent (49/54) of the SCC were found in patients aged more than 40 years and was more common in males than females, a ratio of 1.5:1. Figure 4 shows that 9% (5/54) of the SCC patients were below 41 years of age. The mean age of patients with SCC was 57.9 years. Fifty percent of the patients that presented with SCC were alcohol and tobacco users. Metastatic adenocarcinoma from the prostate gland to the mandible was seen in one patient.

**Figure 5:**
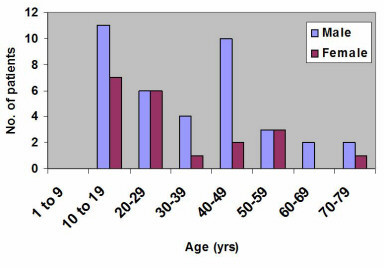
Age distribution of patients with ameloblastoma.

Fifty-eight (93.6%) of the benign odontogenic tumours were diagnosed as ameloblastoma which constituted 34% of all lesions seen during the period under observation. Fifty-one percent (30/58) of the ameloblastoma were found in patients aged between 11 and 30 years as shown in Figure 5. Thirty-six percent (62/171) of all tumours were odontogenic. The mean age of patients with ameloblastoma was 33.5 years. The other benign odontogenic tumours recorded were odontoma, ameloblastic fibroma and myxofibroma.

Non-odontogenic tumours and tumour-like lesions found were fibro-osseous lesions, giant cell granuloma, pyogenic granuloma, salivary pleomorphic adenoma, granular cell leiomyoma of tongue, osteoma, fibroma and lipoma.

Fibro-osseous lesion was diagnosed in sixteen patients (51.6%) eleven of whom had ossifying fibroma, four had fibrous dysplasia and one, cemento-ossifying fibroma. More than 60% of these patients had large, disfiguring lesions. Table 3 is the classification of all the tumours and tumour-like lesions seen during the period of study.

**Table 3:** Malignant and benign tumours

**Tumour**		**No. (%)**
**A.**	**Malignant**	
	Squamous cell carcinoma	54 (62)
	Lymphoma	15 (19)
	Adenocarcinoma	2 (3)
	Chondrosarcoma	2 (3)
	Spindle cell carcinoma	2 (3)
	Mucoepidermoid carcinoma	1 (1)
	Ameloblastic carcinoma	1 (1)
	Plasmacytoma	1 (1)

	**Total**	**78**

		
**B.**	**Benign odontogenic tumours**	
	Ameloblastoma	58 (93.6)
	Odontoma	2 (3.2)
	Ameloblastic fibroma	1 (1.6)
	Myxofibroma	1 (1.6)

	**Total**	**62**

		
**C.**	**Benign non-odontogenic tumours and tumour-like lesions**	
	Fibro-osseous lesions	16 (52)
	Giant cell granuloma	4 (13)
	Pyogenic granuloma	3 (10)
	Salivary pleomorphic adenoma	3 (10)
	Granular cell leiomyoma of tongue	2 (6)
	Osteoma	1 (3)
	Fibroma	1 (3)
	Lipoma	1 (3)

	**Total**	**31**

Unilateral submandibular and cervical lymph node enlargement was common in the malignant lesions. In three patients the lymph nodes were bilateral and in two which were unilateral, the submandibular nodes were fixed.

## Discussion

Tumours and tumour-like lesions affecting the lower face are fairly common in the West African sub-region. Studies in Nigeria [8,9], have also shown that these lesions are common in both adults and children and are mainly found in the mandible. Furthermore, benign as well as malignant lesions are found in the lower face.

We have shown in this retrospective study carried out at the primary Teaching Hospital in Accra, Ghana which receives referrals predominantly from health institutions in the whole of Ghana and neighbouring countries that the frequency of malignant tumours is similar to that of malignant tumours reported in our earlier study of tumours of the midface [11].

Malignant lesions usually found in the lower face include sarcomas of soft and hard connective tissue, carcinomas of the salivary glands, with SCC accounting for more than 90% of reported malignant tumours of the oral cavity, and also melanomas and those that metastasize from distant sites such as the breast, lungs, abdominal organs or even the prostate gland [3-5,12]. Malignant tumours of the jaws are grouped into central and secondary lesions [13]. Central, as originating within the jaw bone and secondary, predominantly oral cancers and metastatic lesions that involve the bone secondarily. The malignant lesions in this study were mainly central or secondary invasion from related tissues and metastasis from distant sites.

The observed SCC prevalence of 69% in this study is closer to the 73.1% found in Zimbabwe [14] but much lower than 90% prevalence reported by Sapp [3] and Neville [4]. SCC was formerly thought to be a tumour with a common presentation in the older age group. However, recent studies report the increasing occurrence of this tumour in the younger age group [15]. Detection of SCC in the younger age group from our retrospective study, supports the current thinking that SCC is no more a tumour only found in the aged but in all adult age groups. A comparison with the results of our earlier studies of midface tumours in Ghana [11] indicates that SCC seems to affect the lower face more than the midface (69% versus 51%). These figures, however, contrast with the overall incidence of 80% SCC detected in the aero-digestive tract as reported by Lund [16].

More than 50% of subjects with SCC either consumed alcohol or smoked tobacco; this is comparable with 45% in the Zimbabwe study [14] admitting such habits. These habits have been described as risk factors for SCC [15,17] and thus may have played a role in the high SCC prevalence seen in this study. Smoking by women in Ghana is not a common practice. However smoke inhalation by women from passive smoking and that from fuel wood may be linked to SCC seen in women. Further studies will help to clarify the situation.

The clinical presentation of the other nonsquamous cell malignant lesions, their treatment and prognosis is known to be highly variable and dependent on the size of tumour, its histologic variant and grade, and extent of spread at the time of biopsy [5]. Nineteen percent of patients with lymphoma is higher than 4.7% found in Zimbabwe [14]. The clinical presentation of loose and displaced teeth as common features and age range of 4 to 12 years detected in Burkitt's lymphoma are similar to those detected by Bruce et al in their study on this malignant tumour, also in Ghana [18].

The incidence of metastatic adenocarcinoma from the prostate gland to the mandible is similar to what has been reported by Dequanter and Laski [19,20]. Primary central mucoepidermoid carcinoma of the jaws is a rare lesion [21] often manifesting as a low-grade lesion composed of well-differentiated mucous and epidermoid cells forming cystic spaces, but invading adjacent tissues without encapsulation. It is usually associated with salivary glands and account for 5–10% of all salivary gland tumours [22]. Similarly, Chondrosarcoma (CS) is rare in the head and neck region and accounts for less than 2% [23] of malignancies in this region. This is consistent with the 3% found in this study, thus confirming that it is a rare tumour. Detection of other rare malignant tumours such as ameloblastic carcinoma, mucoepidermoid carcinoma and plasmacytoma in this study is very interesting and should raise some concerns about these tumours in Ghana and the West African sub-region. Further surveillance studies on tumours from other parts of the country need to be carried out to ascertain the prevalence of these rare malignant tumours in Ghana.

The detection rate of 36.3% benign tumours found to be odontogenic in this study is comparable with that of 32% reported by Adebayo et al in Nigeria [24], but contrasts study from Zimbabwe of 8.6% [25]. Other studies have reported odontoma [10,26,27], or odontogenic myxoma [28] as the most common odontogenic tumours contrary to the findings of ameloblastoma predominating in this study. It is however consistent with Nigerian studies in which ameloblastoma is the most common benign odontogenic tumour seen in children and adults [8,24,28] and also in the study from Zimbabwe in which ameloblastoma comprised 79.1% of odontogenic tumours [25]. The predilection of this tumour for the lower face is in agreement with what has been observed in studies from Nigeria [9,29], Jordan [10] and Zimbabwe [30]. Our previous study of the midface [11] did not find any ameloblastoma which contrasts with that of Al-Khateeb et al [10] who found ameloblastoma in the maxilla in North Jordanian children.

Ameloblastic fibroma, a rare tumour of mixed connective and odontogenic tissue origin is found commonly in young age groups, between 15 and 25 years, with higher prevalence more common in males than females. The tumour presents more frequently in the mandible, usually in the canine to molar region. Radiographically, it appears as unilocular area of radiolucency with smooth outline and it is difficult to distinguish from a unilocular ameloblastoma or dentigerous cyst [31]. Chen et al have reported on the relationship between ameloblastoma and ameloblastic fibroma and suggested that the latter could transform into the former [32].

Ossifying fibroma detected in this study prevalently localized in the mandible is in agreement with what has been described by Lerda et al [33]. It has also been detected in the maxilla, paranasal sinuses and peripheral bones. Its growth is, however, very slow and it is usually asymptomatic, and for the latter reason, it often reaches a considerable size as observed in our patients.

Osteomata of the bones including the jaws, present as bony hard swellings. Patients are known to sometimes develop impacted teeth and diffuse sclerosis of the whole mandible with this condition. These symptoms with intestinal polyposis depicts Gardner's syndrome [34]. Although the patients diagnosed in this study with osteoma of the mandible had bony swellings, they did not exhibit the other symptoms; indicating the possibility of occurrence of solitary osteomas of the mandible.

## Conclusion

Tumours of the lower face comprising the mandible, tongue and adjacent structures are of a diverse group and are fairly common. We have demonstrated in this small study that both malignant and benign tumours and tumour-like lesions are seen in the Ghanaian population. Squamous cell carcinoma and ameloblastoma being the most common malignant and odontogenic benign tumours seen respectively, the two representing more than 65% of all tumours detected during the 8–year study. The odontogenic tumours had a predilection for the mandible. It is evident that the development of certain tumours have a correlation with lifestyle. There is a need to investigate causes of these varied tumours, the prevalence of which cause some concern. We plan carrying out more surveillance studies on tumours from other parts of the country to ascertain the prevalence of the rare malignant tumours in Ghana and West Africa.

## Competing interests

The author(s) declare that they have no competing interests.

## Authors' contributions

**GP **conceived the study, did the literature search, wrote the manuscript and submitted the article.

**GA **analysed data, participated in the literature search and writing of the manuscript

**PA **retrieved the data
